# Job vacancies at the European Centre for Disease Prevention and Control (ECDC)

**DOI:** 10.2807/1560-7917.ES.2023.28.10.2303091

**Published:** 2023-03-09

**Authors:** Eurosurveillance editorial team

**Affiliations:** 1 European Centre for Disease Prevention and Control (ECDC), Stockholm, Sweden

The European Centre for Disease Prevention and Control (ECDC) invites applications for the following positions:

 

**Expert Mathematical Modelling **
**Principal Expert Mathematical Modelling **
The application deadline expires on 17 Apr 2023.

 

For more information, please visit the ECDC website: https://www.ecdc.europa.eu/en/vacancies

